# QM-symex, update of the QM-sym database with excited state information for 173 kilo molecules

**DOI:** 10.1038/s41597-020-00746-1

**Published:** 2020-11-18

**Authors:** Jiechun Liang, Shuqian Ye, Tianshu Dai, Ziyue Zha, Yuechen Gao, Xi Zhu

**Affiliations:** 1 Shenzhen Institute of Artificial Intelligence and Robotics for Society (AIRS), 13-15F, Tower G2, Xinghe World, Rd Yabao, Longgang District, Shenzhen, Guangdong 518172 China; 2 Department of Mathematics, College of Letters and Science, University of California, Santa Barbara 522 University RD, Santa Barbara, CA 93106-3080 USA

**Keywords:** Organic chemistry, Excited states

## Abstract

In the research field of material science, quantum chemistry database plays an indispensable role in determining the structure and properties of new material molecules and in deep learning in this field. A new quantum chemistry database, the QM-sym, has been set up in our previous work. The QM-sym is an open-access database focusing on transition states, energy, and orbital symmetry. In this work, we put forward the QM-symex with 173-kilo molecules. Each organic molecular in the QM-symex combines with the *C*_*n*_*h* symmetry composite and contains the information of the first ten singlet and triplet transitions, including energy, wavelength, orbital symmetry, oscillator strength, and other quasi-molecular properties. QM-symex serves as a benchmark for quantum chemical machine learning models that can be effectively used to train new models of excited states in the quantum chemistry region as well as contribute to further development of the green energy revolution and materials discovery.

## Abstract (summary)

**Table:** 

Measurement(s)	Excitation • molecular structure data • Chemical Properties
Technology Type(s)	quantum chemistry computational method
Factor Type(s)	molecule

Machine-accessible metadata file describing the reported data: 10.6084/m9.figshare.13089554

## Background & Summary

The past few decades have witnessed the construction of various quantum chemical databases such as GDB-13^1^, QM7^2^, QM7b^3^, and QM9^4^. This databases report molecular structure and several energy-related properties, including entropy and band gap. GDB-13 lists 970 million synthetic organic molecules and contains up to 13 heavy atoms, while the QM7 database provides the coulomb matrix and atomization energy of 7165 organic molecules for the GDB-13 subset containing 7 heavy atoms. QM7b extends 13 additional properties of QM7, such as energies of the highest occupied molecular orbital (HOMO) and the lowest unoccupied molecular orbital (LUMO), polarization, and excitation energy of 7,211 organic molecules. Montavon *et al*.^3^ used these databases to train multi-task deep neural networks, using coulomb matrices as descriptors to predict these additional attributes with reasonable accuracy. The most widely used one is the QM9 dataset constructed by Von Lilienfeld *et al*., which contains up to 9 heavy atoms and has ground state geometry, dipole moment, polarimetry, enthalpy, and free energy of approximately 134k molecules, which is popular in the field of artificial intelligence chemistry (AIC).

The excited-state properties of molecules are of great value in practical applications—for example, photosensitizers, phosphorescent molecular probes, and photodynamic therapy (PDT). However, most current open-access databases fail to provide sufficient information on excited-state properties. The fomous QM8^5,6^ contains TD-DFT and CC2 level of electron spectra informations, but some exact transition information such as oscillator strength, transition energy, or transition symmetry is still missing. In recent decades, many discussions and studies on singlet fission have been raised. Singlet fission (SF), with its induced energy conversion process capable of exceeding the traditional Shockey-Queisser limit^7^, enables a singlet exciton to split into two triplet excitons, and is regarded to be capable of improving the efficiency of current photovoltaics. Previous researchers have demonstrated various designs for SF photovoltaics^8–10^, while the development of appropriate SF materials is hindered by limited SF structure database. By involving excited state information, our quantum chemistry database can reveal the development trend of compound properties and guide the rational design of new materials.

Another vital application of accelerated development is artificial intelligence. Checking the excited-state properties of each molecule experimentally is time and energy consuming, and thus the use of quantum mechanical computation (QM) or machine learning algorithm (ML) is necessary in enabling scholars to study the structure and properties of material molecules more efficiently^11–17^ and to compile large databases. However, quantum mechanical computation and machine learning algorithms, especially neural networks, are able to come up with relatively good performance only if large databases are utilized in training and debugging models. To tackle this problem, QM-symex provides an efficient training and evaluation database for data-driven machine learning models in quantum chemistry. Given the information of the first ten singlet states and triplet states, the database has more application value than the original database in terms of the correlation characteristics of orbital symmetry, such as excitation degeneracy and selection rules of transition. This symmetric database can provide additional benefits by allowing researchers to understand and discover structural properties from ML perspectives, eventually make essential contributions to discovering chemical relationships and the synthesis of new organic materials by strong fitting and classification.

What is more, the study of excited molecules is of great importance to the industrialization of renewable resources. Solar energy is one of the essential renewable energy sources and the day-night cycle on earth makes the storage of solar energy an essential prerequisite for solar energy research and utilization. Under the current circumstance, high cost of inorganic materials widely used to store solar energy makes it difficult to realize large-scale commercialization of solar energy. Our research makes it possible to lower the cost of storing solar energy by substuting inorganic materials with the organic ones. In fact, for organic molecules, due to the corresponding relationship between the excited state and the quasi-particle condition, the transition of electrons in different molecular orbitals will lead to many vital phenomena, such as photochromism and fluorescence. More importantly, information on the excited state of the molecule contributes to energy generation. Organic molecules with its low-cost, easy-to-process, and regulated characteristics provide an ideal target for the next generation of the photon industry. So far, much work has focused on the discovery of excited states and corresponding data^18^, including the study of organic photoelectric sensing materials and the study of excited states and photochemistry of organic molecules.

## Methods

In this work, we propose a quantum chemical symmetric excited state database (QM-symex)^19^ that contains 173k molecules. Each organic molecule in QM-symex has been combined with *C*_*n*_*h* symmetry composites^20^. Each molecule in QM-symex contains information about the first ten Singlet transitions and Triplet transitions, including energy, wavelength, symmetry, oscillator strength, spin, and other excimer properties.

**Fig. 1:**
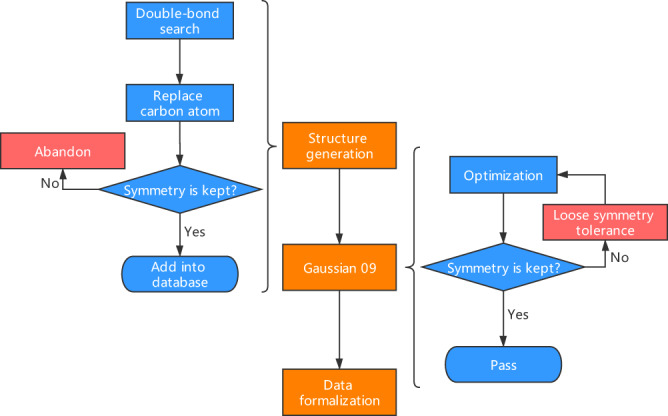
The overall process of the QM-symex generation. The middle part is the mainstream, the left part shows the process of new molecules generation, and the right part is the validation through Gaussian 09.

To prepare the database for better machine learning performance, another 38k molecule is generated in addition to the 135k molecules from QM-sym. We do not use double-bonded carbon atoms at the center in new molecules to ensure the stability. Initially, we decide which symmetry to be used, generate an initial carbon chain, and choose whether to lengthen the side chain or replace the hydrogen atom with halogens. This lengthening and replacement process are also forced to keep the original symmetry. The optimization is performed with 100 cycles to guarantee the minimum energy and stable position. To check whether the molecule still has the original symmetry after optimization, we use a validation step in the Gaussian09 for each cycle. When the position of atoms reaches the restrictions, we will lose the symmetry tolerance and check whether the symmetry is kept overall. If the symmetry is broke, this molecule will be abandoned. For the whole 173k molecules, we choose *Nstates* = 10 and keep the B3LYP/6-31 G level of theory with *Symm* = *VeryLoose* to calculate the first ten transition states. The overall process of database generalization is shown in Fig. 1.

**Fig. 2:**
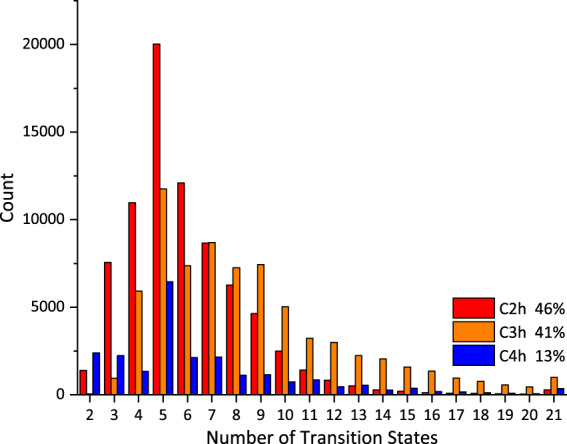
Number of states in the first transition. All of the molecules are included. The legend shows the symmetry component of QM-symex. The lower scheme is the number of transition states versus the count of molecules. Most of the molecules have less than ten states inside the first transition. The colors of both diagrams have the same meaning. Red, orange and blue colors denote *C*_2_*h*, *C*_3_*h*, and *C*_4_*h* molecules.

All the excited state information is extracted from the output from Gaussian09, and is collect into correlated xyz file. Now the QM-symex database is publicly available on figshare (see the code availability section below). It now includes 173k molecular structures (QM_symex_i.xyz) and all properties information in QM-sym, and we add the information of the first ten singlet and triplet transition, including energy, wavelength, orbital symmetry, transition distance, and other quasi-molecular properties. Details about the available properties are recorded in the README file and Table 1 below. Figure 2 shows the overall composition of QM-symex. With 38k new molecules, the *C*_2_*h* occupies 46% capacity and *C*_3_*h*, *C*_4_*h* occupy 41% and 13%, and most of the molecules have three to nine states in the first transition, as shown in Fig. 2.

**Table 1:** Properties from Gaussian09 calculation.

No.	Property	Unit	Description
1	*TS*	/	Transition symmetry group
2	*E*_*t*_	eV	Transition energy
3	λ	nm	Wavelength
4	*f*	/	Oscillator strength
5	*S*^2^	/	Spin

The previous properties from Qm-sym were not listed here. This table shows the properties below xyz coordinates.

## Data Records

All the xyz file is contained in a package on figshare^19^.The xyz file contains the atomic coordinates and the prediction attribute information from the Gaussian 09 calculation; Each structure is indexed as QM_symex_i.xyz, where i is the index of the ordered structure in the database. The xyz file format is one of the most widely used file formats in molecular chemistry, and the structure can be visualized using many free software programs like VESTA. Table 2 presents the basic outline of the xyz format. *N* is the symmetry of the molecule *C*_*N*_*h*, and *n*_*a*_ is the number of atoms in each symmetry unit. The format of the first 3 + *N*·*n*_*a*_ lines is the same as QM-sym with the same ID. Starting from the 4 + *N*·*n*_*a*_ is the transition information. The 4 + *N*·*n*_*a*_ line shows the position of HOMO, for instance, “71”. The following ten lines are the ten transitions. In each line, the information is shown in two-part. The first part is the Singlet transitions and followed by Triplet transitions. The format is: “transition number | (Singlet Part) transition symmetry, energy (eV), wavelength (nm), oscillator strength, spin | (first state) origin orbital, target orbital, probability | (second state) … | (Triplet part) transition symmetry, energy (eV), wavelength (nm), spin | (first state) origin orbital, target orbital, probability | (second state) …”. Take an example as follows: “4|EU 3.9319 315.33 0.0045 0.000|338 341 0.70100|EG 3.8932 318.46 0.0000 2.000|335 344 −0.13252|335 345 −0.12772|340 342 0.59427|340 343 0.21201” from QM_symex_210542.xyz. “4” means it is the fourth transition. “EU,” “EG” are the symmetry of Singlet and Triplet transition. “3.9319” and “3.8932” are the energy of both transitions in eV. “315.33” and “318.46” are wavelengths in nm. “0.0045” and “0.0000” are oscillator strengths, and the following “0.000” and “2.000” are the spin. The next blocks show all the transition states. In this example, the fourth Singlet transition has only one state, and the fourth Triplet transition has four states. “338 341 0.70100” means that this state is from the 338^th^ orbital to the 341^st^ orbital with a probability of 0.701. It is not a problem to determine the correlation between the number-denoted orbitals because the position of HOMO will be denoted in a previous line, as shown in Table 2. That line tells that the number of HOMO is 340, so this transition is from HOMO-2 to LUMO. The format information is also available in the README file.

**Table 2:** xyz file formats for molecular structure and properties.

Line	Content
1, 3 + *N*·*n*_*a*_	Original QM-sym data (properties and xyz coordinates)
4 + *N*·*n*_*a*_	Position of HOMO
5 + *N*·*n*_*a*_	Information of the first Singlet/Triplet transition
…	…
14 + *N*·*n*_*a*_	Information of the tenth Singlet/Triplet transition

The first to the 3 + *N*·*n*_*a*_ lines have the same format as QM-sym. Started from 4 + *N*·*n*_*a*_ are the lines for excited states information. The detailed format is discussed above in the main body.

## Technical Validation

The properties recorded in this database are numerically derived from the DFT calculation and TD-DFT calculation after optimization of molecules with symmetry tolerance and 10^−5^ eV energy convergency. The detailed properties are listed in Table 1. These new molecules are first calculated with B3LYP/6-31 G(2df,p) level in Gaussian 09^21^, and strictly follow the geometry check^20^. The benchmark with the G4^22^, G4MP2^23^, and CBS-QB3^24^ is also processed and is shown in Table 3. Inside the parenthesis are the data recorded in the QM9 database. The maximum number of heavy atoms for molecules in QM9 is 9, but in QM-symex, the number of heavy atoms can reach 60, so a slightly larger error in benchmarks is reasonable and tolerable.

**Table 3:** Benchmark comparison of atomization enthalpies.

Reference	MAE	maxAE	RMSE
CBS-QB3	4.9 (4.5)	6.9 (5.5)	15.4 (13.4)
G4	5.6 (4.9)	6.6 (5.9)	16.4 (14.4)
G4MP2	6.4 (5.0)	8.0 (6.1)	17.6 (16.0)

The data inside the parenthesis are data from QM9, and the other one is the difference comparing to calculated data under B3LYP/6-31 G(2df,p) level.

**Fig. 3:**
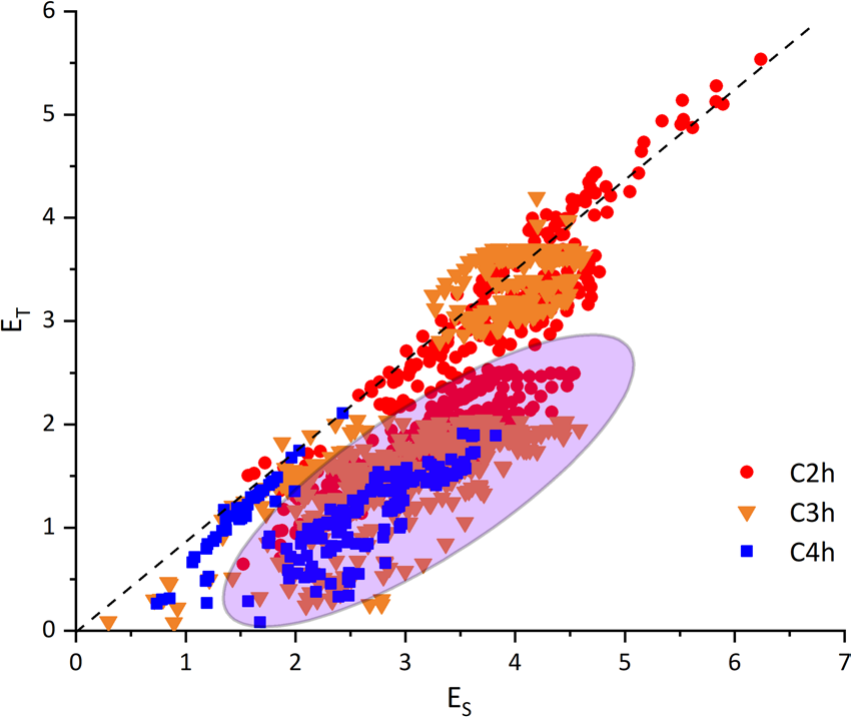
Singlet transition energy versus Triplet transition energy of random-selected molecules. The red dot, orange triangle, and blue square denote molecules with *C*_2_*h*, *C*_3_*h*, and *C*_4_*h* symmetry. From these molecules, we can see two tendencies. The black dash line denotes the first one, which is *E*_*S*_ ≅ *E*_*T*_. The second one is denoted by the purple oval, which is *E*_*S*_ ≅ 2*E*_*T*_ and accords with SF condition.

Based on a simple Molecular orbital picture^25^, the energy difference between singlet and triplet excited states depends on the exchange integral of HOMO and LUMO, which can be characterized by the wave function distance. The exchange integral will nearly vanish if the distance between HOMO and LUMO wave function is demonstrated to be significant. As a result, the singlet and triplet excited state will have equal excitation energy, which implies the first tendency *E*_*S*_ ≅ *E*_*T*_ in Fig. 3. This can also be verified through the calculation of oscillator strength. For structures satisfying *E*_*S*_ ≅ *E*_*T*_, the singlet oscillator strength tends to approach zero value even if it is symmetry allowed. The second tendency acts as a boundary to distinguish potential SF structures and is rounded by a purple oval in Fig. 3. These molecules satisfy *E*_*S*_ ≅ 2*E*_*T*_ and have a large amount, which is helpful to SF-correlated research, and corresponding AI approaches.

**Fig. 4:**
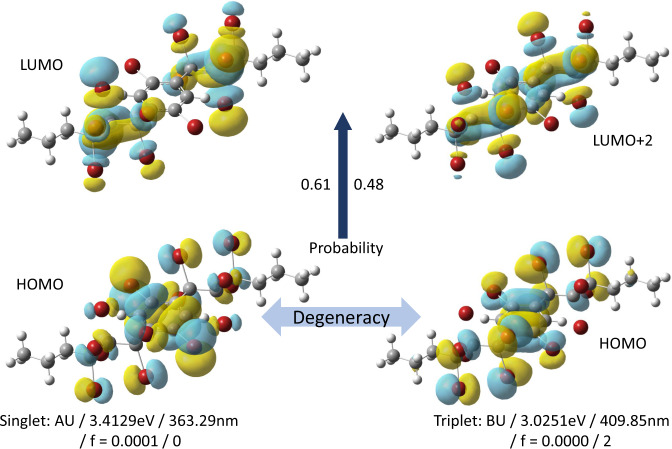
Example of the first transition of QM-symex No.001550. The left part is the Singlet transition, and the right part is the Triplet transition. The symmetry, energy, wavelength, oscillator strength, and *S*^2^ of Singlet transition are AU, 3.4129 eV, 363.29 nm, 0.0001, and 0. For Triplet transition, they become BU, 3.0251 eV, 409.85 nm, 0, and 2. The Singlet transition is from the HOMO to LUMO, and the Triplet transition is from the HOMO (degeneracy = 2) to the LUMO +2. On either hand of the middle upward arrow are two probabilities for Singlet transition state (0.61) and Triplet transition state (0.48). Here 0.61 and 0.48 are the highest probabilities over the Singlet and Triplet states.

For the structure in Fig. 4, we show an example of singlet and triplet transitions on the same molecule. The degeneracy of HOMO is 2, so the two orbitals in the lower half can be considered the same due to the same symmetry. The enormous probabilities of the first Singlet transition and Triplet transition are from HOMO to LUMO and from HOMO to LUMO +2, which are 0.61 and 0.48, as shown in the middle part. The symmetry, energy, wavelength, oscillator strength, and spin of these two transitions are also shown in the lower part of Fig. 4. As is shown in the spin density cloud, the wavefunction of HOMO mainly focuses on the benzene ring, while the wavefunction of LUMO is localized around the halogen atom, resulting in a low exchange integral. Thus, the oscillator strength for singlet excited state is small, and the singlet excited energy is merely larger than triplet excited energy. The triplet excited state is classically forbidden; hence the oscillator strength for triplet is precisely zero.

Nowadays, neural network method can predict the quantum chemical properties with high accuracy; the deep learning community desires a new baseline to evaluate the performance of their method. As far as we know, QM-symex is the first database to provide excited-state data with symmetrical molecules, together with other properties, such as energy, heat capacity, and band gap, in both K and R spaces. Compared with the most widely used QM database QM9, QM-symex raises a higher requirement for the method. The number of data needed to be predicted is not fixed but depends on the number of orbits in different molecules. The neural networks should be capable to output different results according to the input molecules. With the excited-states data provided by QM-symex, researchers can encode the correlation between orbital information and quantum chemical properties in their methods to enhance the accuracy in both excited state and quantum chemical properties prediction.

To demonstrate the potential of deep learning method in predicting excited state, we have run SY-GNN^26^ on this database; the overall classification error for symmetry prediction is 17.01%, and the detailed data are shown in Table 4. The mean-absolute-error (MAE) for vibration frequency prediction is 12.03, and the root-mean-square error (RMSE) 11.81. For vibration mass prediction, MAE is 2.63, RMSE is 4.46. We can see that the accuracy of LUMO classification and HOMO classification are the highest in the conduction band and valence band. Classification accuracy of the neural network drops with the energy difference from HOMO and LUMO. The deep learning method can easily predict the low orbit, but reveals difficulty in explaining high orbit behaviors. This result shows that the calculation and prediction of the error inside the neural network favor the low-energy transition, which is consistent with the theory. The lower accuracy from the orbitals away from HOMO and LUMO shows that further analysis is needed in the neural network to reach higher accuracy on orbital property predictions.

**Table 4:** The prediction classification error (CE) for vibration symmetry on different orbits.

Orbital	LUMO	LUMO +1	LUMO +2	LUMO +3	LUMO +4	LUMO +5
CE	2.50%	8.38%	9.63%	12.66%	18.71%	22.76%
**Orbital**	**HOMO**	**HOMO −1**	**HOMO −2**	**HOMO −3**	**HOMO −4**	**HOMO −5**
CE	2.59%	7.90%	15.95%	24.04%	27.97%	29.20%

## Data Availability

The newest version of QM-symex is available on figshare (10.6084/m9.figshare.12815276).
